# Psychosocial work experience and salutogenic health among nurses and physicians in a reorganized tertiary care hospital: differences and associations

**DOI:** 10.3389/fpubh.2026.1974029

**Published:** 2026-09-30

**Authors:** Jurgita Andruškienė, Aurelija Podlipskytė, Marija Truš, Jūratė Grubliauskienė

**Affiliations:** Faculty of Health Sciences, Klaipėda University, Klaipėda, Lithuania

**Keywords:** nurses, physicians, salutogenic health indicators, SHIS, WEMS, work experience

## Abstract

**Aim:**

The aim of this study was to analyse psychosocial work experience and salutogenic health among nurses and physicians in a reorganized tertiary care hospital in Lithuania.

**Material and methods:**

In 2025, an anonymous hard-copy survey was conducted in a large tertiary care hospital in Lithuania. Ethical approval was obtained from the local bioethics committee. In total 900 questionnaires were distributed to physicians and nurses with 497 returned (55.2% overall response), including different response rates by group (56.0% physicians vs. 54.6% nurses). Each participant completed a three-part questionnaire comprising the Work Experience Measurement Scale (WEMS) rated on a six-point Likert scale, the Salutogenic Health Indicator Scale (SHIS) and demographic questions (sex, age group <40/40–54/≥55, position, years employed in the hospital <4/4–10/>10, and other employment). Indices were standardized to 0–100, internal consistency was assessed with Cronbach’s alpha, group differences were tested using independent-samples t-tests, multiple linear regression was used to model SHIS as the dependent variable from the six WEMS sub-indices (adjusted for age), with all analyses performed in SPSS 19.0 at *p* = 0.05.

**Results:**

The study results showed satisfactory to excellent reliability for the measures, with SHIS (12 semantic differential items on recent affective experiences such as alert–tired and happy–depressed) reaching high internal consistency (Cronbach’s *α* = 0.95). Across the work-experience scale, Likert-type sub-dimensions also showed acceptable to excellent reliability, including supportive working conditions (*α* = 0.91), internal work experience (*α* = 0.75), autonomy (*α* = 0.87), leadership (*α* = 0.94), and the process of change (*α* = 0.95). The respondent profile differed significantly by work position, including a higher proportion of women among nurses than physicians and differences in age distribution, with most participants aged 40–54 years. Nurses reported significantly higher scores than physicians for working conditions (77.6 ± 17.7 vs. 69.7 ± 20.0), internal work experience (81.1 ± 14.4 vs. 77.8 ± 15.2), time experience (76.1 ± 20.9 vs. 59.6 ± 23.6), and SHIS scores (74.1 ± 19.2 vs. 62.7 ± 17.2), while autonomy and leadership did not differ significantly. Regression analyses showed that, among physicians, supportive working conditions (*β* = 0.32, *p* < 0.001), time experience (*β* = 0.26, *p* < 0.001), and process of change (*β* = 0.16, *p* = 0.039) were significantly and positively associated with SHIS. Among nurses, internal work experience (*β* = 0.13, *p* = 0.020), time experience (*β* = 0.24, *p* < 0.001), and process of change (*β* = 0.43, *p* < 0.001) were significantly and positively associated with SHIS, whereas supportive working conditions were not significantly associated (*β* = 0.07, *p* = 0.252). Model explanatory power was R2 = 0.451 for physicians, 0.512 for nurses, and 0.508 for the total sample.

**Conclusion:**

The study instruments showed satisfactory to excellent internal consistency, supporting the credibility of the subsequent comparisons and regression analyses. Nurses reported more favorable psychosocial work experiences and higher salutogenic health than physicians. Salutogenic health was positively associated with supportive working conditions, time experience, and the process of change among physicians, and with internal work experience, time experience, and the process of change among nurses.

## 1 Introduction

Adopting salutogenesis can deepen understanding of how work environments relate to health. While public health and clinical approaches have often treated environments primarily as sources of pathogens, threats, or risks to be eliminated, salutogenesis supports a complementary perspective: environments can also provide enabling conditions, resources that help individuals cope with life challenges and sustain quality of life (1). This is especially relevant in medicine (2), where work is not merely a setting but a major determinant of health through psychosocial conditions such as demands, support, autonomy, recognition, role clarity, and meaning.

Within health systems, nurses and physicians occupy different professional roles, with distinct responsibilities, training pathways, daily workflows, power relations and exposure to teamwork practices. These differences plausibly shape both psychosocial work experiences and salutogenic indicators, including sense of coherence and related health-promoting resources (3). Nurses play a pivotal role in prevention and health promotion, not only by supporting patients’ healthier behaviours, but also by contributing to the broader organizational culture that enables health creation (4). At the same time, salutogenesis emphasizes that individuals’ health-related capacities are shaped by both situational factors and structural conditions at the organizational and societal levels. Yet, some studies using salutogenic concepts have tended to focus more on individual-level salutogenic factors than on how workplace structures and professional contexts contribute to health outcomes (5, 6).

This gap is particularly salient in settings where the health environment is changing or being built. Reorganized tertiary care hospitals represent a unique and underexplored context: work structures, hierarchies, communication pathways, routines, and interprofessional collaboration are still developing, and these conditions can strongly influence psychosocial experiences and health-related resource building. Understanding how nurses and physicians differ in their work experience and salutogenic health indicators in such a setting is therefore important for both theory and practice.

Investigating work experience alongside salutogenic health indicators is crucial because it can inform evidence-based strategies to strengthen health-promoting working conditions (7). Such strategies can enhance not only staff well-being and retention, but also the quality and safety of patient care (8). Moreover, a differential analysis between professional groups can help identify where organizational support, leadership practices, or interprofessional structures may need redesign to foster comprehensibility, manageability, and meaningfulness as core components of sense of coherence.

The aim of this study was to analyse psychosocial work experience and salutogenic health among nurses and physicians in a reorganized tertiary care hospital in Lithuania.

## 2 Materials and methods

### 2.1 Sample

The survey was conducted in 2025 at one of the largest hospitals in Lithuania. Its structure includes departments of internal medicine, obstetrics, surgery, rehabilitation, emergency care, palliative care, psychiatric care, radiology, and the pathology and laboratory medicine centres. The hospital is led by an executive team headed by a hospital director, supported by deputy directors responsible for key areas, including medical services, nursing/care, and administration/operations. Clinical departments and support services are organized under these directorates to deliver care across both settings.

The investigated facility was established in 2023 as a centre for research and medical advancement, providing tertiary-level medical services. The merger aimed to pool expertise, strengthen specialization, ensure more rational allocation of human and material resources, and integrate scientific and international experience into treatment and nursing processes, thereby improving the effectiveness of care, treatment, and rehabilitation for the regional population.

During the study period, a total of 1,350 physicians and nurses were employed across the hospital (831 nurses and 519 physicians). Participants were recruited based on predefined inclusion criteria: physicians and nurses were invited to participate if they worked directly with patients in clinical units. Staff from the following units were excluded from recruitment: the laboratory medicine and blood bank center, the medical genetics center, the pathology center, and the coordination center for the donation of tissues and organs from deceased adults.

After applying the inclusion criteria, the target study population comprised 900 employees (500 nurses and 400 physicians), corresponding to 66.7% of the total number of eligible physicians and nurses. The distribution of nurses and physicians within the study population was determined by the higher number of nurses in the hospital and by the study aim to obtain comparably sized participant groups. Ethical approval was obtained from the local bioethics committee. The survey was administered anonymously using paper questionnaires distributed to participants. Questionnaires were completed by 273 nurses (response rate: 54.6%) and 224 physicians (response rate: 56.0%). The observed response rates were lower than anticipated. This may have been attributable to the hospital’s recent establishment, during which staff may have been adapting to evolving routines and staffing structures, potentially reducing both willingness and opportunity to complete the survey.

The anonymous design helped minimize social desirability and reporting bias. Content validity was supported by the questionnaire’s prior use in earlier studies (9, 10) in comparable hospital settings.

### 2.2 Questionnaires

Each participant completed a three-part questionnaire comprising: the Work Experience Measurement Scale (WEMS) (9); the Salutogenic Health Indicator Scale (SHIS) (10); and demographic questions.

The WEMS includes 32 statements across six dimensions (sub-indices): supportive working conditions (7 items), internal work experiences (6 items), autonomy (4 items), time experience (3 items), leadership (6 items), and process of change (6 items). Items are rated on a six-point Likert-type scale (6 = “totally agree” to 1 = “totally disagree”; Table 1). Total WEMS scores may be computed as a single index ranging from 32 to 192 or separated into the six sub-indices. Sub-index scores are standardized to a 0–100 scale.

**Table 1:** Presentation of the indexes used in the study.

Index	Number of statements	Scale type	Examples of statements	Cronbach´s alpha
SHIS	12	Semantic differential	In the last 4 weeks, I have… felt alert/tired, exhausted; felt happy, optimistic/depressed, sad; felt well/sick.	0.95
Supportive working conditions (WEMS)	7	Likert-type scale	We encourage and support each other at work. I get feedback on the work I do.	0.91
Internal work experience (WEMS)	6	Likert-type scale	I feel that my work is meaningful. I am happy when I go to work.	0.75
Autonomy (WEMS)	4	Likert-type scale	I decide when to do the various work tasks. I decide how to do my work.	0.87
Time experience (WEMS)	3	Likert-type scale	I have enough time during my normal working hours to do my job without time pressure (stress).	0.78
Leadership (WEMS)	6	Likert-type scale	My boss is available when I need him/her. My boss helps us divide our work in a fair way.	0.94
Process of change (WEMS)	6	Likert-type scale	We all had an active part in the process of change. I was well informed about the process of change.	0.95

The SHIS assesses twelve aspects of health, including feeling alert, feeling calm/relaxed, and sleeping well. The SHIS contains one overall question referring to the previous four weeks (“How have you been feeling during the past 4 weeks? The last 4 weeks I have …”), with response categories ranging between positively and negatively worded alternatives (e.g., “felt calm/relaxed” to “felt worried/tense”; Table 1). Responses use a six-step semantic differential with one positive and one negative endpoint. SHIS was used as a single index, with scores ranging from 12 to 72.

Demographic information was collected on sex, age (three groups: <40, 40–54, and ≥55), position, years of employment in the hospital (three groups: <4, 4–10, and >10), and other employment (work beyond the hospital, e.g., private practice and consultancies). These variables have been used in prior studies published elsewhere (11).

### 2.3 Statistical analyses

Total indices of SHIS and WEMS, as well as the six WEMS sub-indices, were standardized so that the scores ranged from 0 to 100. This standardization facilitated comparison of the index distributions. Internal consistency was assessed using Cronbach’s alpha (CA); higher values are generally preferable, although lower values can still be interpreted (12).

For numeric variables, group differences were evaluated using independent-samples t-tests when comparing two groups. To examine the relationship between SHIS and WEMS, multiple linear regression analyses were performed, with SHIS as the dependent variable and the six WEMS sub-indices entered simultaneously as independent variables; the models were adjusted for age. Separate models for physicians and nurses were presented to describe within-group patterns of association and were not intended as formal tests of differences in regression coefficients between professional groups. No profession-by-WEMS interaction terms were tested.

All analyses were performed in SPSS version 19.0. Statistical significance was set at 0.05.

## 3 Results

### 3.1 Psychometric properties of study measures

Table 1 summarizes the psychometric indices used in the study, including the number of statements, the response format (semantic differential or Likert-type), and representative item content for each construct. The SHIS subscale (12 items) employed a semantic differential scale capturing recent affective experiences (e.g., alert–tired, happy–depressed), demonstrating high internal consistency (Cronbach’s *α* = 0.95). Across the WEMS-related dimensions, Likert-type items assessed perceptions of supportive working conditions (*α* = 0.91), internal work experience (*α* = 0.75), autonomy (*α* = 0.87), time experience (*α* = 0.78), leadership (*α* = 0.94), and the process of change (*α* = 0.95). Overall, the reported Cronbach’s alpha values indicate satisfactory to excellent reliability for nearly all measures, supporting their use for subsequent analysis (Table 1).

### 3.2 Sociodemographic and work-related characteristics of respondents

Table 2 summarizes the sociodemographic and work-related characteristics of respondents in the overall study group (*n* = 497) and stratified by work position. A significant gender distribution difference was observed between nurses and physicians (*p* < 0.001), with women constituting 93.0% of nurses and 55.4% of physicians (overall 76.1%). The age categories also differed significantly by work position (*p* < 0.001); however, the overall distribution indicates a majority of respondents were aged 40–54 years (36.0%), followed by those >54 years (31.4%) and <40 years (32.6%). Years of employment in the hospital varied significantly between groups (*p* < 0.001), with >10 years accounting for most nurses (64.5%) and physicians (44.2%) (overall 55.3%). Concurrent employment elsewhere differed significantly by work position (*p* < 0.001), with nurses more often reporting no other employment (76.9%) compared with physicians (40.6%) (overall 60.6% no). The reported effect sizes suggest small-to-moderate differences between nurses and physicians for age (0.160), years of hospital employment (0.210), and gender (0.439), and a small overall magnitude for concurrent employment (0.370).

**Table 2:** Characteristics of the respondents of the total study group and in relation to work position (%).

Characteristics	Nurses (*n* = 273)	Physicians (*n* = 224)	Total study group (*n* = 497)	*p*	Effect size
Gender				<0.001	0.439
Male	19 (7.0)	100 (44.6)	119 (23.9)		
Female	254 (93.0)	124 (55.4)	378 (76.1)		
Age (years)				<0.001	0.160
< 40	71 (26.0)	91 (40.9)	162 (32.6)		
40–54	104 (38.1)	75 (33.5)	179 (36.0)		
> 54	98 (35.9)	58 (25.9)	156 (31.4)		
Years of employment in the hospital				<0.001	0.210
< 4	43 (15.8)	66 (29.5)	109 (21.9)		
4-10	54 (19.8)	59 (26.3)	113 (22.7)		
> 10	176 (64.5)	99 (44.2)	275 (55.3)		
Other concurrent employment				<0.001	0.370
No	210 (76.9)	91 (40.6)	301 (60.6)		
Yes	63 (23.1)	133 (59.4)	196 (39.4)		

Bold values indicate statistically significant results (*p* < 0.05).

### 3.3 Descriptive comparison of WEMS and SHIS scores by work position

Table 3 presents mean values with standard deviations for the WEMS sub-indices, total WEMS, and SHIS by work position (nurses: *n* = 273; physicians: *n* = 224). Nurses reported significantly higher scores than physicians for supportive working conditions (77.6 ± 17.7 vs. 69.7 ± 20.0; *t* = 4.70, *p* < 0.001), internal work experience (81.1 ± 14.4 vs77.8 ± 15.2; *t* = 2.52, *p* = 0.012), time experience (76.1 ± 20.9 vs. 59.6 ± 23.6; *t* = 8.23, *p* < 0.001), and SHIS (74.1 ± 19.2 vs. 62.7 ± 17.2; *t* = 6.29, *p* < 0.001). In contrast, autonomy did not differ significantly between nurses and physicians (66.8 ± 24.7 vs. 69.4 ± 19.4; *t* = −1.30, *p* = 0.192). Leadership also showed no statistically significant between-group difference (76.4 ± 22.2 vs. 77.9 ± 21.2; *t* = −0.80, *p* = 0.424). The process of change was marginal and did not reach conventional significance (69.6 ± 25.5 65.9 ± 22.9; *t* = 1.71, *p* = 0.087). Overall, nurses demonstrated higher total WEMS scores than physicians (75.0 ± 15.8 vs. 71.1 ± 15.6; *t* = 2.82, *p* = 0.005), indicating stronger work-experience perceptions associated with nursing positions.

**Table 3:** Mean values (SD) for the WEMS sub-indices, total WEMS and SHIS in relation to work position.

WEMS sub-indices	Nurses (*n* = 273)	Physicians (*n* = 224)	*t* test	*p*-value	Effect size
Supportive working conditions	77.6 ± 17.7	69.7 ± 20.0	4.70	**<0.001**	0.424
Internal work experience	81.1 ± 14.4	77.8 ± 15.2	2.52	**0.012**	0.228
Autonomy	66.8 ± 24.7	69.4 ± 19.4	-1.30	0.192	0.118
Time experience	76.1 ± 20.9	59.6 ± 23.6	8.23	**<0.001**	0.742
Leadership	76.4 ± 22.2	77.9 ±21.2	-0.80	**0.424**	0.072
Process of change	69.6 ± 25.5	65.9 ± 22.9	1.71	**0.087**	0.155
WEMS total	75.0 ± 15.8	71.1 ± 15.6	2.82	**0.005**	0.254
SHIS	74.1 ± 19.2	62.7 ± 17.2	6.29	**<0.001**	0.624

Bold values indicate statistically significant results (*p* < 0.05).

### 3.4 Associations between SHIS and WEMS sub-dimensions

Table 4 reports results from linear regression models examining associations between the SHIS index and WEMS sub-indices among physicians, nurses, and in the total sample. For physicians, supportive working conditions (*β* = 0.32, *p* < 0.001), time experience (*β* = 0.26, *p* < 0.001), and process of change (*β* = 0.16, *p* = 0.039) were significantly and positively associated with SHIS, whereas leadership showed a negative but non-significant association (*β* = −0.13, *p* = 0.106). Internal work experience and autonomy were not significantly associated with SHIS among physicians (*β* = 0.12, *p* = 0.097; *β* = 0.09, *p* = 0.147). Among nurses, internal work experience (*β* = 0.13, *p* = 0.020), time experience (*β* = 0.24, *p* < 0.001), and process of change (*β* = 0.43, *p* < 0.001) were significantly and positively associated with SHIS, whereas supportive working conditions (*β* = 0.07, *p* = 0.252), autonomy (*β* = 0.01, *p* = 0.864), and leadership (*β* = −0.05, *p* = 0.535) were not significantly associated. Across all employees, time experience (*β* = 0.31, *p* < 0.001) and process of change (*β* = 0.31, *p* < 0.001) supportive working conditions (*β* = 0.19, *p* < 0.001), and internal work experience (*β* = 0.12, *p* = 0.004) were significantly associated with SHIS. Differences in statistical significance between the stratified models were not interpreted as evidence of statistically significant differences between professions, because formal interaction tests were not performed. The models explained a comparable amount of variance, with R^2^ values of 0.512 for physicians, 0.451 for nurses, and 0.508 for the total sample.

**Table 4:** Results from linear regression models among employees of different professions and in total, with SHIS index as the dependent variable and WEMS sub-indices entered as explanatory variables.

	Physicians		Nurses		Total	
WEMS sub-indices	*β*-coeff.	*P*	*β*-coeff.	*p*	β-coeff.	*P*
Supportive working conditions	0.32	**<0.001**	0.07	0.252	0.19	**<0.001**
Internal work experience	0.12	0.097	0.13	**0.020**	0.12	**0.004**
Autonomy	0.09	0.147	0.01	0.864	0.01	0.694
Time experience	0.26	**<0.001**	0.24	**<0.001**	0.31	**<0.001**
Leadership	-0.13	0.106	-0.05	0.535	-0.09	0.078
Process of change	0.16	**0.039**	0.43	**<0.001**	0.31	**<0.001**
Model explanation, R^2^	0.451		0.512		0.508	

Bold values indicate statistically significant results (*p* < 0.05).

## 4 Discussion

The present findings can be compared with an earlier Lithuanian hospital survey conducted in 2010 using the same WEMS and SHIS instruments among 811 employees in one of Lithuania’s largest secondary-care hospitals (13). In both the earlier study and the current cross-sectional survey, time experience was associated with SHIS, however, these were separate samples from different hospital settings and should not be interpreted as demonstrating stability of relationships across time. In the current profession-stratified analyses, time experience and process of change were positively associated with SHIS within both physicians and nurses. Supportive working conditions were significantly associated with SHIS among physicians but not among nurses (*β* = 0.07, *p* = 0.252), whereas internal work experience was significantly associated with SHIS among nurses but not among physicians. Because formal profession-by-WEMS interaction tests were not performed, differences in statistical significance between the stratified models should not be interpreted as evidence that the associations differ statistically between professional groups.

Differences between the earlier and current cross-sectional findings may reflect differences in workforce composition, hospital setting, organizational context, or other unmeasured factors rather than temporal change within the same employees. Autonomy and leadership also showed different patterns between the two surveys: in the current study their associations with SHIS were generally weak and non-significant, whereas the 2010 study reported gender- and age-related patterns and an association between autonomy and SHIS in non-nursing and non-physician roles (13). The current models also accounted for a substantial proportion of variance in SHIS. These comparisons are descriptive and contextual, because the surveys did not follow a longitudinal cohort, they cannot establish changes or persistence of associations over time.

These findings are consistent with broader research reporting associations between psychosocial work environments and health-related outcomes. In Lithuania’s healthcare context, the present results are compatible with a salutogenic interpretation in which psychosocial workplace resources are related to health-relevant outcomes. Across studies, favourable work-environment elements, such as managerial support, collegial cooperation, clear job roles, and autonomy, have beenassociated with better well-being, whereas high workload, stress, conflict, and insecurity have been associated withpoorer health indicators (14–16). Consistent with this literature, Klimanskaite et al. (17) summarized evidence linking more favourable psychosocial hospital work environments with better nurse health and salutogenic indicators.

International evidence also links supportive workplace resources with lowerburnout and depressive symptoms and with greater job satisfaction and engagement, although the strength and pattern of these associations vary according to leadership, staffing, workload, available resources, and role clarity (18). Recent evidence from Central and Eastern Europe is particularly relevant. In a cross-sectional study of 509 nurses in a Polish public hospital. Tomaszewska et al. (19) found significant relationships between areas of professional life, including workload, control, values, and fairness and dimensions of job satisfaction. In another cross-sectional study of 789 nurses working in inpatient health care facilities in Poland, nurses’ well-being was correlated with social support (20). Together, these Polish studies reinforce the relevance of professional life and psychosocial work domains to nurses’ job satisfaction and well-being in a regional healthcare context, while their cross-sectional designs, like the present study, do not permit causal inference. Evidence from other regions likewise indicates associations between social support, collegial relationships, work environment, task structure, and nurses’ job satisfaction or well-being (21–23).

The patterns observed in the profession-stratified analyses, together with the descriptive between-group comparisons, identify areas that may warrant organizational attention. Physicians reported lower scores than nurses for supportive working conditions, internal work experience, and time experience, while autonomy and leadership did not differ significantly between the professional groups. In the regression models, supportive working conditions, time experience, and process of change were significantly and positively associated with SHIS among physicians; among nurses, internal work experience, time experience, and process of change were significantly and positively associated with SHIS, while supportive working conditions were not significantly associated (*p* = 0.252). Leadership was negatively but non-significantly associated with SHIS in both physicians and nurses; therefore, these results should not be interpreted as evidence that leadership caused poorer health or that either professional group was dissatisfied with leadership. Nevertheless, the findings support consideration of workplace strategies that strengthen supportive and participatory leadership, communication between managers and healthcare professionals, staff involvement in organizational decisions, and appropriate professional autonomy. Regular opportunities for physicians and nurses to provide feedback about workload, time pressure, leadership practices, organizational change, and workplace relationships may help identify concerns and inform targeted improvements. Because the present quantitative design cannot explain why particular aspects of the work environment were evaluated less favorably or how staff experience leadership in everyday practice, a subsequent qualitative phase using semi-structured interviews or focus groups with physicians and nurses would be valuable. Such a mixed-methods approach could clarify the organizational, interpersonal, and professional mechanisms underlying the quantitative findings and support more specific, context-sensitive recommendations for healthcare workplaces.

Several limitations should be considered when interpreting these findings. First, the overall response rate was 55.2% (56.0% among physicians and 54.6% among nurses), raising the possibility of non-response bias. Because information on non-respondents was not available, we could not determine whether participants differed systematically from eligible staff who did not respond. Second, although the regression models were adjusted for age, residual confounding cannot be excluded. Other measured or unmeasured factors, including sex, years of employment, concurrent employment, clinical unit, workload, and other occupational or personal characteristics, may have influenced the observed associations. Third, WEMS and SHIS were assessed simultaneously using self-reported questionnaires. This common-method approach may have introduced shared-method variance, reporting bias, or response tendencies that could inflate or otherwise affect associations between perceived work experiences and self-reported health indicators. Fourth, the study was conducted in a single reorganized tertiary care hospital in Lithuania; therefore, the findings may not be generalizable to other hospitals, healthcare settings, regions, or health systems with different organizational structures and workforce characteristics. In addition, the cross-sectional design does not establish temporality or causality, and the regression coefficients should be interpreted as associations rather than predictors, determinants, or drivers of SHIS. The profession-stratified regression models were exploratory: formal profession-by-WEMS interaction tests were not performed, and detailed regression diagnostics were not retained for reporting; therefore, subgroup coefficients should be interpreted as within-group associations rather than evidence of statistically significant differences between professions. Comparisons with the 2010 Lithuanian survey involve separate cross-sectional samples and different hospital contexts and therefore indicate only similarities or differences in observed patterns, not stability or change within individuals over time. Future research should prioritize multicentre sampling, longitudinal designs, more comprehensive control of potential confounders, and, where feasible, data from multiple sources or measurement occasions to reduce common-method bias and clarify temporal relationships. A subsequent qualitative phase using semi-structured interviews or focus groups with physicians and nurses could further explore how leadership, autonomy, internal work experience, time pressure, organizational change, and other workplace dimensions are experienced in this reorganized hospital.

## 5 Conclusion

The study instruments demonstrated satisfactory to excellent internal consistency, strengthening the credibility of the subsequent comparisons and regression analyses.

Nurses reported significantly higher scores than physicians for supportive working conditions, internal work experience, time experience, overall WEMS, and SHIS, indicating differences between the professional groups in several dimensions of psychosocial work experience and salutogenic health.

In profession-stratified regression models, higher SHIS scores were significantly associated with supportive working conditions, time experience, and the process of change among physicians, whereas among nurses, higher SHIS scores were significantly associated with internal work experience, time experience, and the process of change. Supportive working conditions were not significantly associated with SHIS among nurses. These profession-specific findings describe within-group associations and should not be interpreted as evidence of statistically significant differences in the strength of associations between physicians and nurses in the absence of formal interaction testing.

In the total sample, supportive working conditions, internal work experience, time experience, and the process of change were significantly and positively associated with SHIS. The regression models explained 45.1% of the variance in SHIS among physicians, 51.2% among nurses, and 50.8% in the total sample.

## Data Availability

The raw data supporting the conclusions of this article will be made available by the authors, without undue reservation.
